# HOW CHILDHOOD NEGLECT AFFECTS LATER LIFE LONELINESS

**DOI:** 10.1093/geroni/igae098.3997

**Published:** 2024-12-31

**Authors:** Karina Tavares, Gina Barbera, Cindy Tsotsoros

**Affiliations:** University of Rhode Island, Kingston, Rhode Island, United States; University of Rhode Island, Kingston, Rhode Island, United States; University of Rhode Island, Kingston, Rhode Island, United States

## Abstract

Individuals who experience neglect within the first eighteen years of life are at increased risk for loneliness later in life. This study explores whether the type of neglect experienced (emotional, physical, or both), and the age at which it occurs, affects later-life loneliness. This was accomplished by using a subsample of 169 women aged 18−86 who reported experiencing at least one form of neglect. Demographic indicators, the 17-item ACEs questionnaire (Felitti et al., 1998), and the UCLA Loneliness Scale (Russell, 1996) were examined. Neglect was categorized into three groups: emotional neglect, physical neglect, and both emotional and physical neglect. Age of first experienced neglect was categorized into three groups: ages 0−5, 6−11, and 12−18 years. When comparing the three categories of neglect, an ANOVA revealed that those who experience both emotional and physical neglect experience the highest levels of later-life loneliness. A multiple linear regression model showed that age alone predicted 2.3% of the variance in loneliness. When including both age and neglect, the model accounted for 9.8% of the variance, and adding their interaction improved the prediction to 12.3%. This study underscores that experiencing both emotional and physical neglect, particularly during early childhood, significantly increases the risk of loneliness in later life.

